# Outcomes of percutaneous vertebroplasty in multiple myeloma: a tertiary neurosciences experience with long-term follow-up

**DOI:** 10.3389/fonc.2024.1291055

**Published:** 2024-04-11

**Authors:** Hannah E. Holmes, Vartan Balian, Saminderjit Kular, Ruth Batty, Daniel J. A. Connolly, Andrew Chantry, Andrew Martin

**Affiliations:** ^1^Radiology Department, Sheffield Teaching Hospitals National Health Service (NHS) Foundation Trust, Sheffield, United Kingdom; ^2^Radiology Department, The University of Sheffield, Sheffield, United Kingdom; ^3^Radiology Department, Newcastle upon Tyne Hospitals NHS Foundation Trust, Newcastle upon Tyne, United Kingdom

**Keywords:** vertebroplasty, multiple myeloma, vertebral compression fracture (VCF), vertebral augmentation, Visual Analogue Scale (VAS), Oswestry Disability Index (ODI)

## Abstract

**Background:**

Multiple myeloma is diagnosed in 5,800 people in the United Kingdom (UK) each year with up to 64% having vertebral compression fractures at the time of diagnosis. Painful vertebral compression fractures can be of significant detriment to patients’ quality of life. Percutaneous vertebroplasty aims to provide long-term pain relief and stabilize fractured vertebrae.

**Methods and materials:**

Data was collected from all cases of percutaneous vertebroplasty performed on patients with multiple myeloma from November 2017 to January 2019. Pain scores were measured using the Visual Analogue Scale (VAS) and Oswestry Disability Index (ODI) pre-procedure, 2 months post procedure and 4 years post-procedure. Procedure related complications and analgesia use were also documented.

**Results:**

22 patients were included with a total of 119 vertebrae treated. Patients reported a significant improvement in overall pain score with a median pre-procedure VAS of 8 and a median post-procedure VAS of 3.5 (p<0.0001). There was a median pre-procedure ODI score of 60% and a median post-procedure ODI score of 36% (p<0000.1). There was improvement across all ODI domains and a 77% reduction in analgesic requirement. There were small cement leaks into paravertebral veins or endplates at 15 levels (12%) which were asymptomatic. There were 8 responders to the long-term follow-up questionnaire at 4 years. This demonstrated an overall stable degree of pain relief in responders with a median VAS of 3.5 and median ODI of 30%.

**Conclusion:**

At this center, vertebroplasty has been shown to reduce both VAS and ODI pain scores and reduce analgesia requirements in patients with VCFs secondary to multiple myeloma with long lasting relief at 4 years post-procedure.

## Introduction

Multiple myeloma is a plasma cell tumor characterized by osteolytic bone lesions, pathological fractures, and hypercalcemia. Painful vertebral compression fractures (VCFs) caused by multiple myeloma significantly affect the quality of life of patients. A total of 5,800 people in UK are diagnosed with multiple myeloma each year (1), with 34%–64% having vertebral compression fractures at the time of diagnosis (2). The complications of vertebral compression fractures in multiple myeloma include reduced quality of life due to difficulty in completing activities of daily living (ADLs), respiratory problems, and gastric problems with proven increased morbidity and mortality (3, 4). The majority of patients with VCFs will require a high-dose opioid pain relief to manage symptoms.

Percutaneous vertebroplasty is a management option in which polymethylmethacrylate is injected into the affected vertebrae (5). This is believed to structurally stabilize the vertebral body and denature nerve endings. There is an exothermic reaction associated with the cement setting in the vertebral body, and the heat generated is thought to cause damage to pain perception nerves.

Regarding vertebroplasty, in general, there is a degree of disagreement in the literature with several previous randomized clinical trials investigating the use of vertebroplasty in acute osteoporotic fractures finding no significant benefit (6–8). There are yet to be any randomized controlled trials regarding the use of vertebroplasty specifically in VCFs caused by multiple myeloma. However, multiple previous studies of the effectiveness of vertebroplasty specifically in multiple myeloma have been more supportive (9–12). One paper demonstrated that 82% and 89% of patients had a significant improvement in subjective rest pain and activity pain, respectively (11). Another demonstrated a reduction in median Visual Analogue Scale (VAS) from a score of 9 pre-procedure to a score of 1 at 3 months post-procedure (12). Previous studies presenting longer-term follow-up have not followed patients beyond 18 months post-procedure.

The authors aim to use data to demonstrate the outcomes from this tertiary center to assess whether our outcomes are consistent with other centers and to find out more about the effects of vertebroplasty on pain control after 18 months. Additionally, the data can be presented to patients to support fully informed consent and help select patients who are most likely to benefit.

## Methods

### Institutional approval

This retrospective service evaluation did not require research ethics committee approval. The project was registered with the local audit and service evaluation team. All data were handled in accordance with local information governance policies.

### Study design and case selection

All eligible patients undergoing percutaneous vertebroplasty for vertebral compression fractures secondary to multiple myeloma between November 2017 and January 2019 were included. The data was collected prospectively. All procedures were performed by one of two consultant neuroradiologists in a single tertiary neurosciences center using the Medtronic Kyphon vertebroplasty kit.

Outcome was measured using two pain scores, VAS and Oswestry Disability Index (ODI) (13). The ODI is a questionnaire which assesses the degree of disability due to pain, with a score of 0–5 across 10 domains which include sitting, standing, personal care, sleeping, and social life. The overall score is often expressed as a percentage. The VAS is a psychometric device shown to be statistically measurable and reproducible and can be used to assess disease-related symptom severity usually but not specifically regarding pain. The patients score their overall current pain using an image in a scale of 1–10.

Each patient completed a pre-procedural VAS and ODI and a follow-up VAS and ODI at 2 months post-procedure. In the case of a treatment requiring more than one sitting, the follow-up VAS and ODI assessment was performed immediately prior to the next treatment sitting. The pre- and post-procedure scores were then analyzed with a *t*-test used to determine significance. Where multiple sittings were undertaken, the second and third pre-procedure scores were taken as the post-procedure score for the previous sitting. Data on complications was also collected. Analgesic requirement was categorized using the WHO analgesic ladder, and subsequent changes in the dose of analgesia or changes in the category of analgesia were recorded. A long-term follow-up ODI and VAS was sent to surviving patients at 4 years post-procedure. The data was analyzed using Microsoft Excel (version 2312) and GraphPad Prism (version 10.2.1). Median scores, mean percentage change, 95% confidence intervals, and *t*-test (to obtain *p*-values) were calculated, and graphs were created using the GraphPad Prism software.

#### Diagnostic assessment

When a clinician suspects VCF in a known multiple myeloma patient, an MRI of the whole spine should be performed to investigate abnormal areas of signal and vertebral fractures. Typical sequences at this center include sagittal T1 and T2 sequences of the whole spine with axial T1 and T2 through any abnormal levels, with the number and location of these slices decided by the performing radiographer and supervised by a neuroradiologist if required. STIR sequences may be added to identify bone edema, demonstrated as increased signal. Features of an involved vertebra include low vertebral marrow signal on T1-weighted sequences, high signal on STIR weighted sequences, and disruption of the bony cortex of the endplates with loss of vertebral body height.

CT of the involved levels assists in deciding suitability by assessing pedicle involvement and demonstrating bony anatomy. It is important to check that the posterior cortex of the vertebral body is intact or cement is more likely to leak into the spinal canal and cause cord injury.

#### Performing percutaneous vertebroplasty

All patients were reviewed prior to each procedure at a pre-operative clinic by a neuroradiologist. The patients were given an information booklet and were asked to complete a pre-procedural Visual Analogue Score (VAS) and Oswestry Disability Index (ODI) questionnaire. Imaging was reviewed, and the procedure and associated risks were explained to the patient. If the patient was suitable for vertebroplasty, this was offered to the patient and written consent was obtained. The consent procedure involved discussing the most frequent and significant risks which include pain, bleeding, infection, further fracture, nerve damage, cement leak, paralysis, and cement embolus.

The number of levels to be treated was planned; the levels more severely affected or causing the most pain were prioritized. Three or four vertebral bodies can be treated in one sitting, and where the patients required multiple treatments, these were offered in sittings 6–8 weeks apart. Antiplatelets and anticoagulants were stopped 5 days prior to the procedure in accordance with hospital protocol. On the day of the procedure, each patient attended the hematology day ward. Up-to-date FBC and coagulation blood tests were obtained with an anesthetic and neuroradiologist’s clinical review.

**Figure 1:**
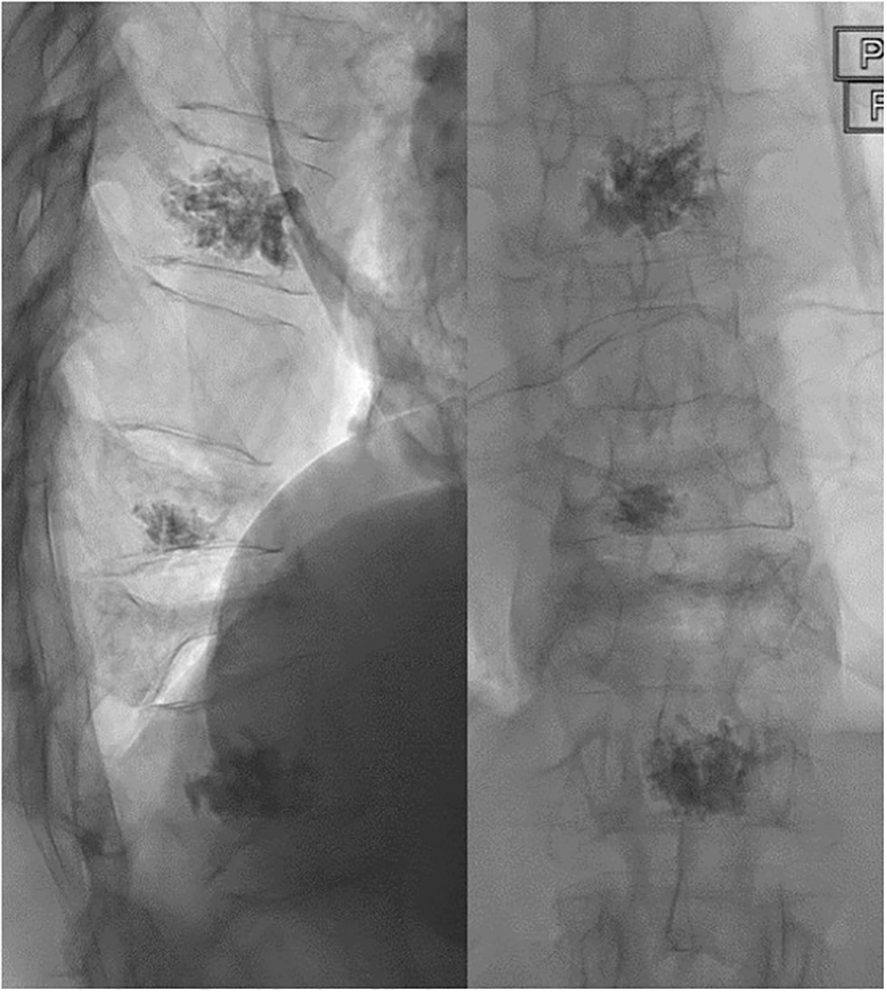
Lateral and anteroposterior fluoroscopic images immediately post-vertebroplasty treatment, acquired at Sheffield Teaching Hospitals. The images demonstrate radio-opaque bone cement in the fractured T9, T11, and L1 vertebral bodies. There is a further fracture of the T12 vertebra with severe collapse.

Vertebroplasty was performed under conscious sedation with the patient in the prone position. Conscious sedation methods varied depending on each individual anesthetist’s preference. The planned levels were treated under fluoroscopic guidance using Medtronic Kyphon vertebroplasty kit. Percutaneous access to the vertebral body was obtained via a bi- or unipedicular approach, depending on the pedicular and vertebral body anatomy. A 10- or 11-gauge Kyphon introducer needle with a beveled tip was used to access the vertebral body. An 11-gauge Kyphon hand drill was used to core out a channel in the vertebral body for the Kyphon cement cannula. Access to all vertebral levels to be treated was obtained before Kyphon Xpede bone cement was slowly injected via the cement cannula under continuous fluoroscopic imaging. Following the procedure, the patient returned to the day unit for observations, with a post-procedure care plan provided. If well, the patient was discharged home on the same day with appropriate advice on aftercare. **Figure 1** demonstrates fluoroscopic appearances after a successful vertebroplasty.

## Results

There were 22 patients included. The median age was 67 years (range: 52–79 years). There were 14 female and eight male patients. A total of 119 levels were treated. the levels treated included T6 to L5. The total number of levels treated per patient ranged from one to 11. A total of 11 (50%) patients were treated in one sitting, five (23%) patients were treated over two sittings, five (23%) patients were treated over three sittings, and one (5%) patient was treated over four sittings.

**Figure 2:**
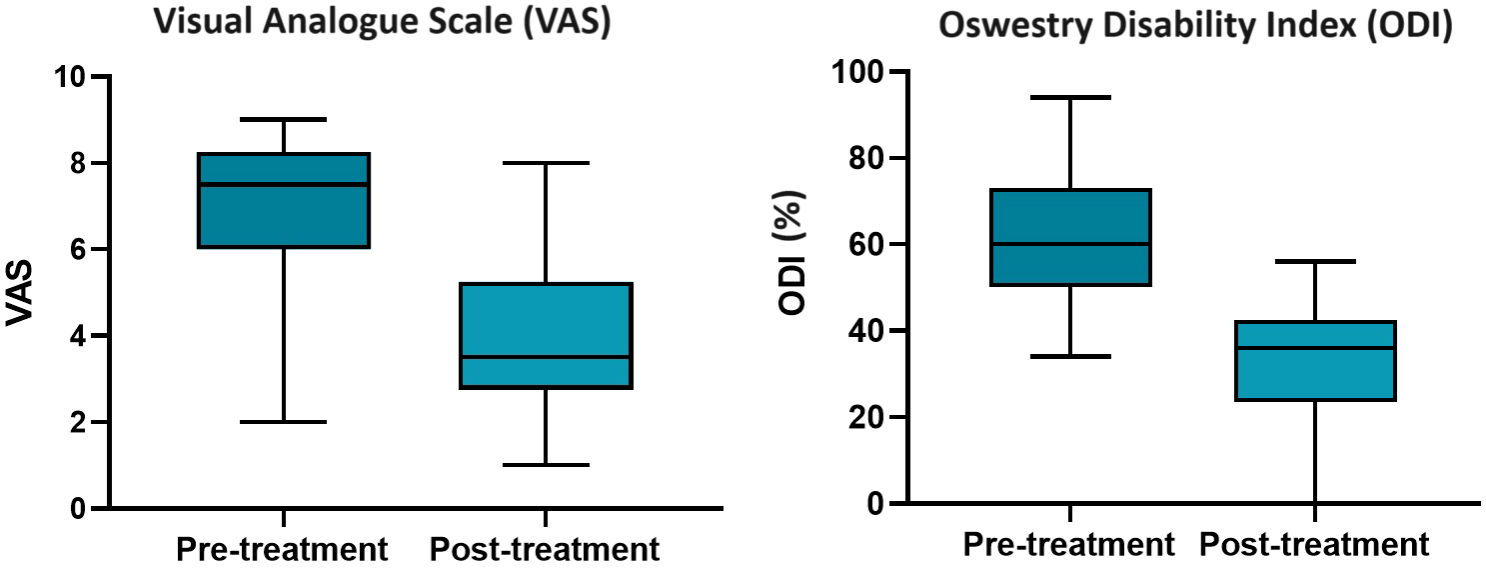
Box and whisker plots demonstrating the median Visual Analogue Scale and Oswestry Disability Index scores pre- and post-treatment, with the median and range indicated by horizontal solid lines.

The VAS pain score was reduced in all patients with a median VAS pre-treatment score of 8 (range: 2–9) and post-treatment score of 3.5 (range: 1–7) (*p* < 0.0001) (**Figure 2**). This was the case for the analysis of pre-procedure and post-first procedure scores and pre-procedure and post-all procedures (where multiple sittings were undertaken).

There was a statistically significant difference between median overall ODI scores before and after treatment in multiple myeloma. The median pre-procedure ODI score was 60% (range: 34%–94%) with a median post-procedure score of 36% (range: 20%–56%) (**Figure 2**). The mean percent reduction from pre- to post-procedure ODI score was 44%.

**Figure 3:**
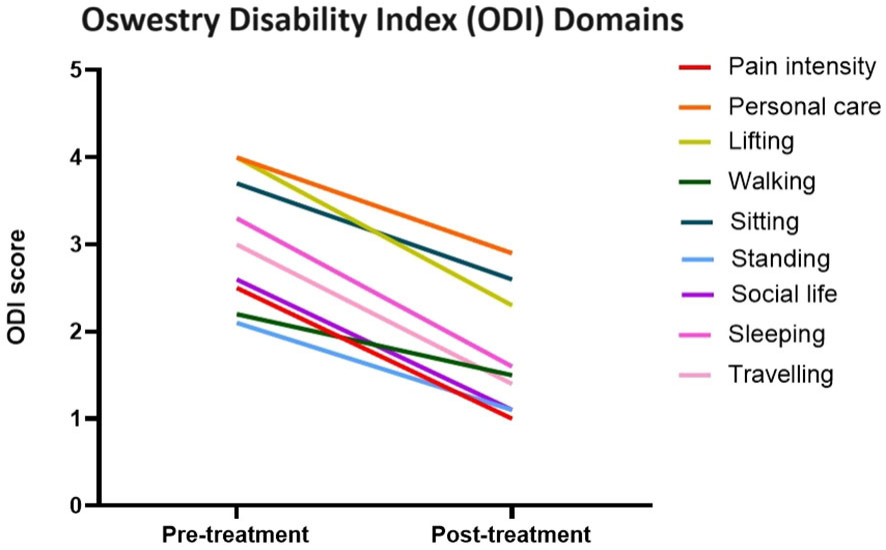
Line graph demonstrating the change in Oswestry Disability Index pain scores across multiple domains pre- and post-treatment.

All domains of the ODI showed improvement post-treatment, with eight out of the 10 domains demonstrating a statistically significant improvement (**Figure 3**). The two domains where the improvement was not deemed statistically significant were sitting and sleeping. However, both domains had a relatively low average pre-procedural ODI of 2 and 2.1, respectively, and so any potential improvement in symptoms and therefore improvement in score would be relatively small.

There was a 77% overall reduction in analgesia requirements across all patients. This includes a reduced dose, reduced strength (step down the WHO analgesic ladder), or no analgesia required at all. For those with a pre-procedure VAS of 8 or more, there was 71% reduction in analgesia requirement, and for those with a pre-procedure VAS of 7 or less, there was 83% reduction.

There were cement leaks at 15 levels (12%), with 12 into paravertebral veins and three into the endplate. There was one incident of cement leak into a paravertebral vein which subsequently caused a pulmonary cement embolus. All patients were asymptomatic from the cement leaks.

**Figure 4:**
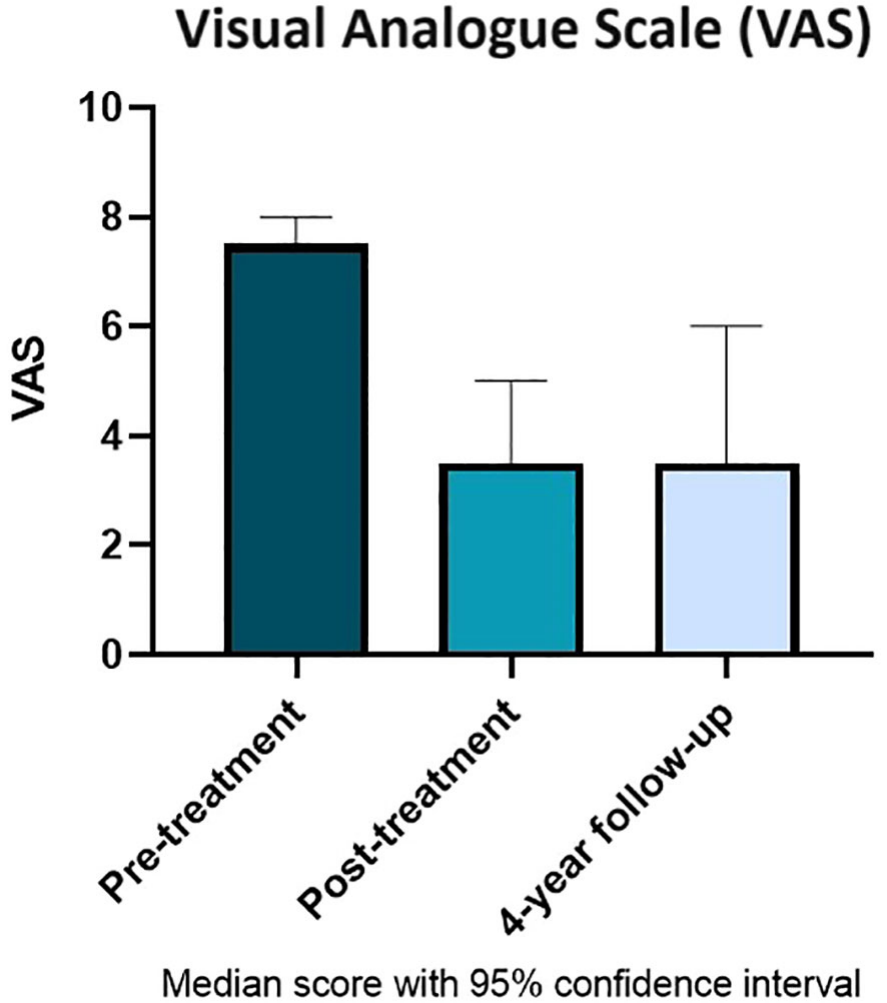
Median Visual Analogue Scale score with 95% confidence intervals pre- and post-treatment compared with 4-year follow-up.

At the 4-year follow-up, there were eight responders and six non-responders. Eight of the 22 patients were deceased at the time of sending out questionnaires, which represent a 36% 4-year mortality rate with a range of time to death after the initial procedure of 64 to 1,588 days. The median VAS at 4-years post-procedure was 3.5 (range: 1–6) with median ODI of 30% (range: 16–60%) (**Figure 4**). The follow-up questionnaires were completed at a median time of 3 years and 11 months.

## Discussion

VCFs in multiple myeloma are secondary to the osteolytic process with subsequent loss of bone stability and collapse of the vertebral body. The aim of vertebroplasty is to provide pain relief in painful VCFs, to improve the quality of life, and to reduce risks associated with immobility and opiate analgesia use. This study reports positive findings with a statistically significant improvement in both VAS and ODI pain scores post-procedure, which is long-lasting and is shown to persist up to 4 years post-procedure.

The most improved ODI individual domain scores were in the “social life” and “personal care” category, with 18 showing an improvement. These categories could be perceived as more subjective than the more physical categories but do have a meaningful contribution to improving the quality of life. There was an improvement for 16 patients in the “lifting”, “walking”, and “traveling” categories. The best improvement appears to be perceived in activities which require the most movement, whereas pain scores during sleeping were more likely to be stable.

The reduction in analgesia requirement post-procedure is a further evidence of the effectiveness of the procedure. The capacity to step down the analgesic ladder benefits the patient by reducing the side effects and risk of opiate-related complications. The WHO analgesic ladder allows for a broad categorization and analysis of the multitude opiate and non-opiate analgesic options. Examples of the changes of analgesic requirements include one patient who required a 100-μg/h fentanyl patch pre-procedure, reduced to only requiring 100 mg of tramadol twice daily post-procedure (reduction of strength, i.e., a step down the analgesic ladder); one patient who required 10 mg of oxycodone four times daily pre-procedure, reduced to 5 mg twice daily post-procedure (reduced dose); and one patient who required a 25-μg/h buprenorphine patch and as-required oromorph, reduced to no regular analgesia post-procedure. There are limitations to this method of analysis due to the breadth of medications in each category, the various mechanisms of action of each analgesic, and the complexities of an individual’s response to each medication type. Patients may also have had analgesic requirements due to pain from other sources, typically myeloma in other parts of the skeleton.

It is worth noting that there was an improvement in analgesia requirement in both those with a VAS of 8–10 and those with a VAS of 7 or less. In fact, there was a greater degree of improvement in those with a VAS of 7 or less. This supports the hypothesis that percutaneous vertebroplasty should be offered to those considered to be in both “severe” and more “moderate” pain.

The responses received at 4-year follow-up survey further support the positive findings with additional evidence that the degree of pain relief is long-lasting. If the procedure is successful in reducing pain initially, most patients can expect that this degree of pain relief will continue for the order of years. To the authors’ knowledge, no other studies have assessed pain outcomes at more than 18 months post-procedure. The mortality rate of 36% at 4 years post-procedure is in line with the UK 5-year survival of 52%, as reported by the office for national statistics (14). This indicates that the vertebroplasty procedure probably does not affect the mortality rate associated with multiple myeloma, although the sample size in this study is not large enough to draw a statistically significant conclusion.

One injection of 119 (0.8%) was complicated by cement embolus. The cement was seen to pass into an adjacent paravertebral vein at the time of injection, and despite cessation of injection, it continued to fill the vein and then broke off. A post-procedure chest radiograph confirmed a cement embolus to a pulmonary vessel in the right upper zone. The patient was asymptomatic from the event, and no further action was required. The documented complication was discussed with the patient following the procedure.

Although this paper reports outcomes for vertebroplasty alone, some centers may also use kyphoplasty alongside vertebroplasty. Kyphoplasty uses a balloon, inserted via the vertebral pedicle into the vertebral body, to expand the collapsed vertebra and create a cavity prior to cement injection. Published data gives some disagreement over whether kyphoplasty achieves better vertebral body height restoration (15, 16). Vertebroplasty alone is preferred in this center as the priority in this cohort of patients is analgesic effect, in addition to consideration regarding time and financial resources.

### Comparison with similar studies

To understand how our techniques and procedure outcomes compare with other centers, we performed a short literature review with the existing publications. Simony et al. presented data from 17 patients with multiple myeloma and treated over 24 sessions. They demonstrated a median preoperative Visual Analogue Scale (VAS) score of 7.6, with improvement observed in all patients. The median VAS pain score decreased to 3.2 at 3-month follow-up. These numbers are comparable to our own findings. There was the same complication rate with cement leakage in 12.5% of the patients, of which all were asymptomatic.

McDonald et al. published a series of 67 multiple myeloma patients treated with vertebroplasty over 8 years. They measured pain using the VAS and Roland-Morris Disability Questionnaire (RDQ) and showed that 82% and 89% of patients experienced a significant improvement in subjective rest pain and activity pain, respectively. They also showed that 65% of patients required fewer narcotics after vertebroplasty and 70% had improved mobility. These results again show a similar improvement when compared to our outcomes.

A more recent paper published by Nas et al. reported outcomes of 166 levels performed on 41 patients with multiple myeloma. They demonstrated median VAS scores of patients which decreased from 9 at 1 day before the procedure to 6 at 1 day after the procedure, to 3 at 1 week after the procedure, and eventually to 1 at 3 months after the procedure. During the procedure, cement leakage was observed at 68 vertebral levels (41%).

## Conclusion

Our data show that percutaneous vertebroplasty in vertebral compression fractures caused by multiple myeloma provides a statistically significant reduction in pain. Reduction in opiate and other analgesia requirements further evidences the reduction in pain and reduces potential opiate complications. Our 4-year follow-up data gives patients the confidence that their pain relief can be long-lasting. We propose that vertebroplasty remains a key element in a comprehensive suite of measures provided to tackle myeloma bone disease.

## Data availability statement

The raw data supporting the conclusions of this article will be made available by the authors, without undue reservation.

## Author contributions

HH: Writing – review & editing, Writing – original draft, Visualization, Project administration, Methodology, Investigation, Funding acquisition, Formal analysis, Data curation, Conceptualization. VB: Writing – original draft, Writing – review & editing, Methodology, Formal analysis, Data curation, Conceptualization. SK: Writing – review & editing, Data curation. RB: Writing – review & editing. DC: Writing – review & editing, Methodology. AC: Writing – review & editing, Visualization. AM: Writing – review & editing, Visualization, Supervision, Resources, Project administration, Methodology, Investigation, Formal analysis, Data curation, Conceptualization.
