# Supplementary material for: Outcomes of percutaneous vertebroplasty in multiple myeloma: a tertiary neurosciences experience with long-term follow-up
Source: Front Oncol. 2024 Apr 11;14:1291055. doi: 10.3389/fonc.2024.1291055 (PMC11044180; doi:10.3389/fonc.2024.1291055)

Supplementary Materials

*Supplementary figure 1: Sagittal T1 and T2 whole spine sequences acquired at Sheffield Teaching Hospitals for the indication of a patient with known multiple myeloma and back pain. Findings: There are fractures of the T8, T11 and L4 vertebral bodies which had progressed since previous imaging. There is also progressive disease at T12, L3 and L5. There is persistent marked flattening of the T7 vertebra. No cord compression, although there is retropulsion of bone fragment at the T11 level.*


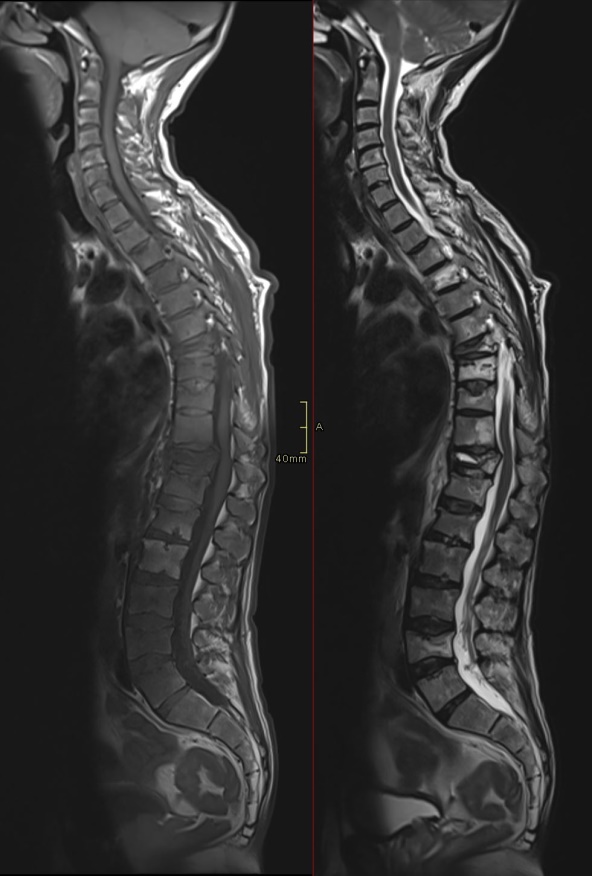


*Supplementary figure 2: Unenhanced CT of the thoracic and lumbar spine performed for vertebroplasty planning, acquired at Sheffield Teaching Hospitals. Axial slice through the T12 vertebral body. There is a vertebral wedge compression fracture of the T12 vertebral body. There is a fracture line extending through the posterior wall.*


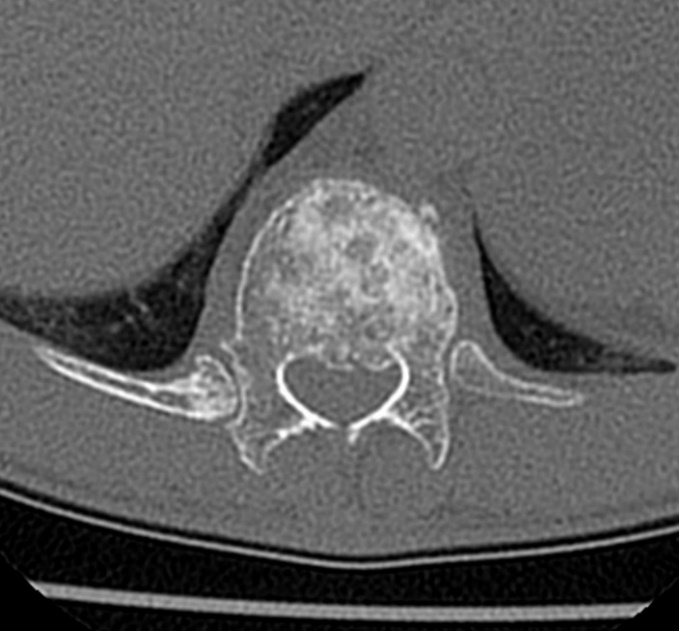


*Supplementary figure 3: A graph demonstrating change in analgesia requirements after vertebroplasty.*


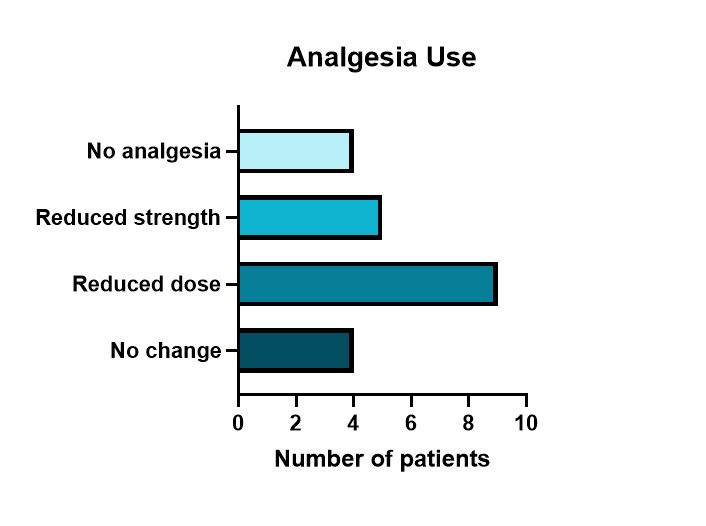


*Supplementary figure 4: PA Chest radiograph acquired in Sheffield Teaching Hospitals. 54-year-old male with a diagnosis of multiple myeloma attended for second sitting of vertebroplasty with treatment to T7, T11, L2 and L4. During injection of T11, cement entered adjacent vein and was seen to break off. The post-procedure chest radiograph confirmed cement embolus in a right upper lobe vessel. The patient was asymptomatic.*


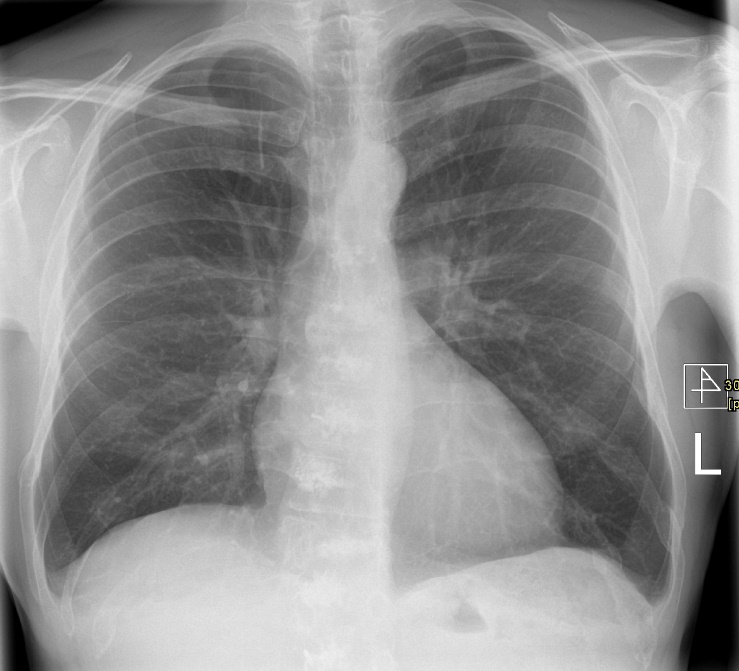

Supplement: Supplementary file 1 [file DataSheet_1.docx]
